# Multiple sclerosis-induced neuropathic pain: pharmacological management and pathophysiological insights from rodent EAE models

**DOI:** 10.1007/s10787-013-0195-3

**Published:** 2013-11-14

**Authors:** Nemat Khan, Maree T. Smith

**Affiliations:** Centre for Integrated Preclinical Drug Development and School of Pharmacy, The University of Queensland, Level 3, Steele Building, St. Lucia Campus, Brisbane, QLD 4072 Australia

**Keywords:** Experimental autoimmune encephalomyelitis, Multiple sclerosis, Neuropathic pain, Allodynia, Neuroinflammation, Pharmacological management

## Abstract

In patients with multiple sclerosis (MS), pain is a frequent and disabling symptom. The prevalence is in the range 29–86 % depending upon the assessment protocols utilised and the definition of pain applied. Neuropathic pain that develops secondary to demyelination, neuroinflammation and axonal damage in the central nervous system is the most distressing and difficult type of pain to treat. Although dysaesthetic extremity pain, L’hermitte’s sign and trigeminal neuralgia are the most common neuropathic pain conditions reported by patients with MS, research directed at gaining insight into the complex mechanisms underpinning the pathobiology of MS-associated neuropathic pain is in its relative infancy. By contrast, there is a wealth of knowledge on the neurobiology of neuropathic pain induced by peripheral nerve injury. To date, the majority of research in the MS field has used rodent models of experimental autoimmune encephalomyelitis (EAE) as these models have many clinical and neuropathological features in common with those observed in patients with MS. However, it is only relatively recently that EAE-rodents have been utilised to investigate the mechanisms contributing to the development and maintenance of MS-associated central neuropathic pain. Importantly, EAE-rodent models exhibit pro-nociceptive behaviours predominantly in the lower extremities (tail and hindlimbs) as seen clinically in patients with MS-neuropathic pain. Herein, we review research to date on the pathophysiological mechanisms underpinning MS-associated neuropathic pain as well as the pharmacological management of this condition. We also identify knowledge gaps to guide future research in this important field.

## Introduction

Multiple sclerosis (MS) is an inflammatory demyelinating disease of the central nervous system (CNS) resulting in motor, sensory and cognitive impairment (Compston and Coles 2008). It is the most common neurological disease in young adults affecting more than 2 million people globally (MSIF 2013). The prevalence and incidence of this disorder is two- to threefold higher in females compared with males (Disanto and Ramagopalan 2013). The pathological hallmark of MS is the lesion, a focally demyelinated area within the CNS white matter and cortex with a variable degree of inflammation, gliosis, axonal loss and incomplete remyelination (Compston and Coles 2008). Although the precise cause of MS is unknown, it is generally regarded as an autoimmune disease that is triggered by environmental factors (Ramagopalan et al. 2010).

There is a tendency towards MS susceptibility for individuals living in temperate climates with a North–South gradient above and below the equator (Herna′n et al. 1999; Hauser 2005). The worldwide geographical distribution of MS (Pugliatti et al. 2002) and the lack of genetic differences between monozygotic twins discordant for MS (Baranzini et al. 2010) substantiate a causal role for environmental factors in its pathophysiology. Epidemiological studies reveal that smoking increases the risk of MS (Herna′n et al. 2005) and that vitamin D may have a protective role in reducing the risk of developing MS (Munger et al. 2004). Pathogens including *Chlamydia pneumonia* (Sriram et al. 1998), human herpes virus-6 (HHV-6) (Soldan et al. 1997) and Epstein-Barr virus (EBV) (Serafini et al. 2007) are possible infectious causes of MS.

Pain is a common disabling symptom of MS. The estimates of MS pain prevalence vary widely in the range 29–86 % (Solaro and Uccelli 2011; O’Connor et al. 2008) depending upon the assessment protocols utilised and the definition of pain being applied. The incidence of chronic pain in MS is not correlated with disease severity (Kalia and O’Connor 2005). Patients may experience nociceptive pain such as muscular cramps (called flexor spasms), leg spasms, headaches and migraine concurrently with neuropathic pain (Thompson et al. 2010). Neuropathic pain is more persistent in nature and is one of the most commonly distressing symptoms experienced by patients even in the early stages of the disease (O’Connor et al. 2008; Thompson et al. 2010). Neuropathic pain associated with MS is inadequately relieved or not relieved at all with conventional analgesics such as non-steroidal anti-inflammatory drugs or opioid analgesics such as morphine (O’Connor et al. 2008; Kalman et al. 2002; Truini et al. 2011). Instead, adjuvant drugs such as the tricyclic antidepressants (TCAs), serotonin and noradrenaline reuptake inhibitors (SNRIs), and anticonvulsants are utilised as first-line drug therapy for alleviation of MS-associated neuropathic pain (Solaro and Uccelli 2011; Truini et al. 2011). However, randomised, double-blind, placebo-controlled trials of these agents are lacking.

## Clinical presentation and prevalence of MS-induced neuropathic pain

Pain associated with MS is heterogeneous in nature encompassing various forms of nociceptive, neuropathic or mixed pain conditions (Truini et al. 2013). Further subdivision into nine categories has been suggested based upon the proposed underpinning mechanisms (Truini et al. 2013). Herein, we have reviewed research on the most common MS-related neuropathic pain conditions including ongoing pain in the extremities (dysaesthetic extremity pain), as well as paroxysmal pain (trigeminal neuralgia and L’hermitte’s phenomenon) (O’Connor et al. 2008; Truini et al. 2013).

Multiple sclerosis-induced neuropathic pain develops as a direct or indirect result of demyelinating lesions in the brain and spinal cord, and therefore is termed “central neuropathic pain” (CNP) (O’Connor et al. 2008; Osterberg and Boivie 2010). Its clinical presentation can also be categorised as stimulus independent or dependent (Osterberg et al. 2005; Svendsen et al. 2005b). The former includes persistent or paroxysmal pain, whereas evoked pain is characterised by hyperalgesia (exaggerated pain response to noxious stimuli) and allodynia (pain response to normally non-noxious stimuli) (Osterberg et al. 2005; Svendsen et al. 2005b).

### Dysaesthetic extremity pain

Dysaesthetic extremity pain is characterised by burning, tingling or aching dysaesthesia predominantly in the legs that may be unilateral or bilateral and is often worse at night (Truini et al. 2011; O’Connor et al. 2008). In patients with MS, dysaesthetic extremity pain is the most commonly reported type of neuropathic pain, having a life-time prevalence of 12–28 % and it is very challenging to treat (Truini et al. 2011; Nurmikko et al. 2010). Clinically, patients with primary progressive or progressive-relapsing MS are more likely to suffer from dysaesthetic CNP, whereas patients with relapsing-remitting disease are less affected (Boneschi et al. 2008; Nurmikko et al. 2010).

### Trigeminal neuralgia

Trigeminal neuralgia (TN) is defined by the International Association for the Study of Pain (IASP) as “sudden, usually unilateral, severe, brief, stabbing recurrent episodes of pain in the distribution of one or more branches of the trigeminal nerve” (Merskey and Bogduk 1994). TN is further divided into two types, classical and symptomatic, as termed by The International Headache Society (Headache Classification Subcommittee of the International Headache 2004). Classic TN has no cause other than neurovascular contact between the trigeminal nerve root and a blood vessel (Zakrzewska and McMillan 2011). However, neuralgia is called symptomatic if it occurs as a consequence of MS or a tumour (benign or malignant) or if it is due to structural abnormalities such as arteriovenous malformations (Zakrzewska and McMillan 2011). The prevalence of trigeminal neuralgia in patients with MS ranges from 2 to 6.3 % (Putzki et al. 2009; Solaro et al. 2004; Osterberg et al. 2005). Although classic TN and MS-associated TN are both characterised by pain, more frequent sensory deficits and occasional bilateral pain that occur in 20 and 7 % of patients, respectively, are useful criteria for differentiating MS-associated TN from classical neuralgia (Truini et al. 2011; Cruccu et al. 2008).

### L’hermitte’s sign

L’hermitte’s phenomenon is described as a transient, paroxysmal electric shock-like discharge, usually evoked by neck flexion which is felt in the back of the neck and spreads to the lower limbs. Although this phenomenon is not an exclusive symptom of MS, it is frequently reported by patients with MS (Nurmikko et al. 2010) with a prevalence ranging from 9 to 41 % (Solaro et al. 2004; Al-Araji and Oger 2005). Although this symptom is typically self-limiting with spontaneous resolution after some weeks, its frequency and intensity may be troublesome in some patients (Nurmikko et al. 2010).

## Pharmacological management of MS-induced neuropathic pain

Evidence-based recommendations on the pharmacological management of neuropathic pain promulgated by the Neuropathic Pain Special Interest Group of the International Association for the Study of Pain (IASP) encompass the treatment of MS-induced CNP (Dworkin et al. 2010). The recommended first-line drug treatments include TCAs (e.g. nortriptyline and amitriptyline), SNRIs (e.g. duloxetine and venlafaxine), voltage-gated calcium channel α2-δ subunit ligands (e.g. gabapentin and pregabalin), and topical lignocaine (Na^+^ channel blocker). Strong opioid analgesics (e.g. morphine, oxycodone, methadone, fentanyl) and tramadol (alone or in combination with a first-line agent) are generally regarded as second-line treatments (Dworkin et al. 2007; Dworkin et al. 2010). Third-line agents that may be used as second-line treatments in some circumstances include other antiepileptic drugs (e.g. carbamazepine, lamotrigine, oxcarbazepine, topiramate, and valproic acid), mexiletine (orally active lignocaine analogue), *N*-methyl-d-aspartate receptor antagonists (e.g. ketamine, memantine) and topical capsaicin (Dworkin et al. 2007; Dworkin et al. 2010).

At present, there are relatively few randomised controlled trials (RCTs) of drug treatments in patients with MS-associated neuropathic pain (Table 1). Hence, its pharmacological management is generally guided by the findings of RCTs conducted in patients with spinal cord injury-induced CNP or peripheral neuropathic pain syndromes (Nurmikko et al. 2010). In the next section, we review clinical studies conducted in patients with MS-associated neuropathic pain. Clinical studies where the pain type was not specified or it was of a non-neuropathic nature were excluded, as were case studies (Table 1).

**Table 1:** Summary of clinical trials for pharmacological management of MS-associated neuropathic pain

Medication/dose range or mean daily dose	Study design	Type of pain assessed; number of patient (*n*)	Pain relief outcome or proportion of patients achieving pain relief	Reference
MS-associated persistent or central neuropathic pain				
Nortriptyline (10–50 mg) or self-applied TENS	Randomised single-blind	Upper extremities pain (*n* = 59)	Both treatments significantly reduced VAS pain scores	Chitsaz et al. (2009)
Lamotrigine (25–400 mg)	Randomised, double-blind, placebo-controlled	CNP (*n* = 12)	Insignificant pain relief above placebo	Breuer et al. (2007)
Levetiracetam (2,000–3,000 mg)	Randomised, single-blind, placebo-controlled	Constant NP (*n* = 13) Intermittent NP (non-trigeminal) (*n* = 5) Mixed (constant/intermittent NP) (*n* = 2)	Significant pain relief	Rossi et al. (2009)
Levetiracetam (3,000 mg)	Randomised, double-blind, placebo-controlled	CNP (*n* = 27)	Overall insignificant pain relief above placebo; patient subgroups reported relief of lancinating pain or absence of touch-evoked pain	Falah et al. (2012)
Lamotrigine (25–400 mg) (add-on therapy)	Randomised, double-blind, placebo-controlled	CNP (*n* = 9)	Insignificant pain relief	Silver et al. (2007)
Dronabinol (10 mg)	Randomised double-blind, placebo-controlled	CNP (*n* = 24)	Significant decrease in median spontaneous pain intensity relative to placebo in last week of treatment	Svendsen et al. (2004)
Dronabinol (10 mg)	Randomised, double-blind, placebo-controlled	CNP (*n* = 24)	Modest analgesic effect	Svendsen et al. (2005a)
Sativex (THC:CBD) (Max 48 sprays per day, each delivering 2.7 mg of THC and 2.5 mg of CBD)	Randomised, double-blind, placebo-controlled, parallel group	CNP Dysaesthetic pain (*n* = 59) Painful spasms (*n* = 7)	Significant pain relief above placebo for mean pain intensity	Rog et al. (2005)
Baclofen (50 μg I.T.)	Randomised, double-blind, placebo-controlled	Patients exhibited mixed chronic dysaesthetic or spasm-related pain (*n* = 4)	Short-term pain relief above placebo for dysaesthetic pain or spasm-related pain	Herman et al. (1992)
Baclofen (5–1,200 μg I.T.) + Morphine (800–9,500 μg I.T.)	Open-label	Dysesthesia or burning sensations mostly exhibited along with spasticity pain (*n* = 9)	Insignificant relief of neuropathic pain by baclofen alone; alleviated only by baclofen in combination with morphine	Sadiq and Poopatana (2007)
Gabapentin (300–2,400 mg)	Open-label	All patients experienced more than one type of pain (neuropathic and non-neuropathic) (*n* = 25)	Pain relief data for individual neuropathic pain symptoms not reported. Overall moderate to excellent pain relief indicated by 15/22 patients. Side effects reported by 11/22.	Houtchens et al. (1997)
Lamotrigine (25–400 mg) (add-on therapy)	Open-label	Continuous limb pain (*n* = 6) Paroxysmal limb pain (*n* = 9) Painful tonic spasms (PTS) (*n* = 8)	Continuous limb pain: no relief in 5/6. Significant relief of paroxysmal limb pain and PST to varying degrees	Cianchetti et al. (1999)
Sativex (THC : CBD) (<8 sprays in 3 h to max of 48 sprays per day)	Open-label	CNP (*n* = 63)	Significant relief with no evidence of tolerance. Adverse effects reported by 92 % of patients	Rog et al. (2007)
Morphine (41 mg)	Non-randomised, single-blind, placebo-controlled	Chronic central pain (non-trigeminal) (*n* = 14)	Effective pain relief in 4/14 patients only after high doses	Kalman et al. (2002)
MS-associated paroxysmal neuropathic pain (Trigeminal neuralgia/L’hermitte’s sign)				
Carbamazepine (760 mg)	Double-blind, placebo-controlled	TN (*n* = 9) Paroxysmal paraesthesia (*n* = 3) Paroxysmal limb pain (*n* = 7)	TN: 4/9 (complete relief) Paroxysmal paraesthesia: 3/3 (complete relief) Paroxysmal limb pain: Partial or no relief	Espir and Millac (1970)
Carbamazepine (dose not reported)	Open-label	TN (*n* = 35)	*n* = 27 10/27 (complete relief) 10/27 (partial relief) 12/27 (subsequently went for surgical treatment)	(Hooge and Redekop 1995)
Oxcarbazepine (600–1,200 mg)	Open-label	Paroxysmal pain (*n* = 12)	9/12 (complete pain relief) 1/12 (incomplete pain relief) 2/12 (discontinued study due to side effects)	Solaro et al. (2007)
Lamotrigine (150–200 mg)	Open-label	TN (*n* = 5)	5/5 (complete relief)	Lunardi et al. (1997)
Lamotrigine (25–400 mg)	Open-label	TN (*n* = 18)	17/18 (complete or nearly complete pain relief)	Leandri et al. (2000)
Carbamazepine (400 mg) + Gabapentin (850 mg) (Gp-1)	Open-label	TN Gp-1 (*n* = 6) Gp-2 (*n* = 5)	Gp-1: 5/6 (complete pain relief) Gp-2: 5/5 (complete pain relief) Significant pain relief by co-administered gabapentin and lamotrigine at lower doses *c.f.* either component alone	Solaro et al. (2000)
Lamotrigine (150 mg) + Gabapentin (780 mg) (Gp-2)				
Gabapentin (600–1,200 mg)	Open-label	TN (*n* = 6) Dysesthesia (*n* = 3) PTS (*n* = 11) Ocular ataxia (*n* = 1)	TN: 5/6 (complete relief) Other symptoms: improved in all patients	Solaro et al. (1998)
Pregabalin (75–300 mg)	Open-label	TN (*n* = 2) Paroxysmal dysaesthesia (*n* = 7) PTS (*n* = 6) Dystonia of upper limbs (*n* = 1)	9/16 (complete relief) 4/16 (partial relief) Efficacy for each pain type not reported	Solaro et al. (2009)
Misoprostol (300–800 μg)	Open-label	TN (*n* = 7)	6/7 (complete or partial pain relief)	Reder and Arnason (1995)
Misoprostol (600 μg)	Open-label	TN (*n* = 18)	8/18 (complete pain relief after 2 weeks)	DMKG study group (2003)
Topiramate (200–300 mg)	Open-label	TN (*n* = 6)	5/6 (complete relief) Pain relief in 6th patient achieved by concomitant use of carbamazepine	Zvartau-Hind et al. (2000)
Topiramate (200–300 mg)	Open-label	TN (*n* = 4) Paroxysmal dysaesthesia (*n* = 4) PTS (*n* = 5)	Incomplete pain relief for all symptoms. Very good pain relief (VAS score ≤3) for TN and paroxysmal pain	D’Aleo et al. (2001)
Lignocaine (6–8.8 mg/kg/h for 30 min followed by maintenance dose of 2–2.8 mg/kg/h Mexiletine (300–400 mg)	Open-label	LH (*n* = 12) PTS (*n* = 10) Itching/pain (*n* = 7) Persistent dysaesthesia (*n* = 15)	LH: 10/12 (resolved with lidocaine); PTS: 7/10 (resolved with lidocaine) 10/10 (relieved with mexiletine) Itching/pain: 7/7 (relieved with lidocaine or mexiletine) Persistent dysaesthesia: Partial relief	Sakurai and Kanazawa (1999)

*CNP* central neuropathic pain, *NP* neuropathic pain, *MS* multiple sclerosis, *VAS* visual analogue scale, *THC* Tetrahydrocannabinol, *TENS* transcutaneous electrical nerve stimulation, *n* number of patients, *CBM* cannabis-based medicine, *CBD* cannabidiol, *TN* trigeminal neuralgia, *LH* L’hermitte’s sign, *Gp* group, *μg* microgram, *mg* milligram, *kg* kilogram, *hr* hour, *PTS* painful tonic spasms, *I.T.* intrathecal

### Treatment of MS-induced persistent neuropathic pain

Although TCAs are recommended as the drugs of choice for management of CNP associated with MS (Truini et al. 2011; Solaro and Uccelli 2011), these agents have many adverse effects such as drowsiness, dry mouth, constipation, hypotension and urinary retention (Mir and Taylor 1997). In a randomised, single-blind clinical trial, the TCA, nortriptyline, was equi-effective with self-applied transcutaneous electrical nerve stimulation for reducing MS-related pain (Table 1). However, nortriptyline produced an array of typical adverse effects associated with TCA administration (Chitsaz et al. 2009). Overall efficacy and optimal dosing regimens of TCAs for the relief of MS-neuropathic pain are lacking as large placebo-controlled RCTs have not been undertaken (Solaro and Uccelli 2011). The mode of action of TCAs and SNRIs for relief of neuropathic pain is attributed to their inhibition of presynaptic reuptake of the biogenic amines, serotonin and noradrenaline, that augment descending inhibitory signalling from the brain to the spinal cord (Coluzzi and Mattia 2005). As selective serotonin reuptake inhibitors (SSRIs) are ineffective for the relief of neuropathic pain (Coluzzi and Mattia 2005), noradrenaline reuptake inhibition is assumed to be the mechanism underpinning the analgesic efficacy of TCAs and SNRIs for relief of peripheral neuropathic pain (Coluzzi and Mattia 2005). However, a recent randomised double-blind, placebo-controlled clinical trial of the SNRI, duloxetine, in patients with spinal cord injury-induced CNP, showed that although duloxetine significantly improved dynamic and cold allodynia relative to placebo, pain intensity was not significantly reduced above placebo (Vranken et al. 2011). Hence, the extent to which SNRIs have efficacy for the relief of MS-induced CNP is unclear.

The efficacy of anticonvulsants including lamotrigine (Cianchetti et al. 1999; Breuer et al. 2007; Silver et al. 2007), levetiracetam (Rossi et al. 2009; Falah et al. 2012) and topiramate (D’Aleo et al. 2001) for the relief of MS-associated persistent or paroxysmal (non-trigeminal) neuropathic pain has been assessed in several small clinical studies (Table 1). In each of these clinical trials, patients reported either incomplete pain relief or limited tolerability (Table 1). Open-label investigation of the efficacy of gabapentin in 25 patients with MS-associated neuropathic pain showed that although patients reported moderate to excellent pain relief (Houtchens et al. 1997), five patients discontinued treatment due to adverse effects including somnolence, insomnia and dyspepsia.

Since discovery of the endogenous cannabinoid system and identification of CB1 and CB2 receptors that are expressed predominantly in the CNS and on peripheral immune cells, respectively, cannabinoids have been investigated for efficacy in neuropathic pain (Rice 2008). Although cannabinoid efficacy for relief of MS-related CNP has been demonstrated (Rog et al. 2005; Rog et al. 2007; Svendsen et al. 2004; Svendsen et al. 2005a), there were many treatment-related adverse effects including dizziness, dry mouth, headache, tiredness or muscle weakness (Rog et al. 2007; Svendsen et al. 2004). Additionally, concern on the risks of acute psychosis and cannabis misuse associated with its therapeutic use (Rice 2008) has restricted these agents to second- or third-line medications for the treatment of MS-associated neuropathic pain (Dworkin et al. 2007; Dworkin et al. 2010; Attal et al. 2010).

In work by others, intravenous morphine was efficacious for the relief of MS-neuropathic pain in a few patients at high doses, consistent with its generally poor efficacy for the relief of other types of neuropathic pain (Kalman et al. 2002). In two small studies, intrathecal baclofen alone (Herman et al. 1992) or in combination with morphine (Sadiq and Poopatana 2007) reportedly alleviated MS-related neuropathic pain. However, baclofen analgesia was short-lived and may have been confounded by concurrent motor impairment (Herman et al. 1992).

### Treatment of MS-induced trigeminal neuralgia (paroxysmal pain)

Carbamazepine is the most effective first-line treatment for MS-associated TN, in a manner similar to classic TN, despite its poor tolerability profile (Solaro and Uccelli 2011). The frequency and severity of carbamazepine side effects often leads to treatment discontinuation (Hooge and Redekop 1995; Espir and Millac 1970) as these side effects including leg muscle weakness and micturition problems can mimic MS relapse (Solaro et al. 2005). Oxcarbazepine, the keto derivative of carbamazepine effectively alleviated TN in nine of twelve patients, but with better tolerability than carbamazepine (Solaro et al. 2007). By combining lower doses of carbamazepine with gabapentin, complete relief of MS-associated TN was achieved with very good tolerability (Solaro et al. 2000). Although there are no randomised, double-blind, placebo-controlled clinical studies of either gabapentin or lamotrigine for the relief of MS-associated TN, several small open-label studies have been published (Table 1). Their findings suggest that gabapentin alone (Solaro et al. 1998), lamotrigine alone (Lunardi et al. 1997; Leandri et al. 2000) or both in combination (Solaro et al. 2000) may have efficacy. Likewise, most patients reported at least partial relief of MS-associated TN in small open-label clinical studies of misoprostol (Reder and Arnason 1995; DMKG study group 2003) and topiramate (Zvartau-Hind et al. 2000; D’Aleo et al. 2001).

### Treatment of MS-induced L’hermitte’s sign (paroxysmal pain)

Anticonvulsants such as carbamazepine, oxcarbazepine and gabapentin are recommended as drug treatments for relentless pain due to L’hermitte’s sign (Truini et al. 2011). Although lignocaine infusion or to lesser extent oral mexiletine alleviated the paroxysmal pain of L’hermitte’s sign in small open-label clinical studies (Sakurai and Kanazawa 1999), neither drug was efficacious for relief of persistent painful symptoms associated with MS (Sakurai and Kanazawa 1999).

From the foregoing, it is clear that there is an urgent unmet medical need for new drug treatments that are highly efficacious and have good tolerability profiles for the relief of MS-associated neuropathic pain. This unmet medical need is driving preclinical research on the pathobiology of MS-associated neuropathic pain as a means for identifying novel “druggable” targets for use in pain therapeutics drug discovery programs.

## Pathophysiology of MS-induced neuropathic pain

In contrast to the wealth of research on the pathophysiology of peripheral nerve injury-induced neuropathic pain in the past two decades, there has been a relative paucity of research on the pathobiology of MS-associated neuropathic pain. The spinothalamic tract is well known to have a key role in the transduction of nociceptive information from the spinal cord to the brain (Willis and Westlund 1997). Patients with MS have abnormal pinprick and temperature sensations (Osterberg et al. 2005; Svendsen et al. 2005b), suggestive of a role for spinothalamic tract dysfunction in the pathobiology of this condition (Osterberg and Boivie 2010), in a manner similar to patients with stroke-induced CNP (Boivie et al. 1989; Vestergaard et al. 1995). However, as a spinothalamic tract lesion alone (without neuronal hyperexcitability) does not induce CNP in patients with spinal cord injury (Finnerup et al. 2003), MS-associated neuropathic pain can be subdivided into (1) primary pain caused by the lesion, and (2) secondary pain due to an indirect consequence of the lesion (O’Connor et al. 2008).

In patients with MS, sodium channel blockers such as lignocaine and mexiletine alleviate positive pain symptoms (dysaesthesia, L’hermitte’s sign and painful tonic seizures), whereas the negative symptoms (paralysis and hypoesthesia) are exacerbated (Sakurai and Kanazawa 1999). These clinical observations suggest that ectopic firing and hyperexcitability of demyelinated sensory neurones mediate the positive symptoms, whereas conduction blockade may underpin the negative symptoms (Sakurai and Kanazawa 1999). Dysaesthetic extremity pain is thought to be due to lesions in the cervical and thoracic regions of the spinal cord resulting in distortion of the sensory modalities associated with ascending spinothalamic nociceptive signal transduction and/or impaired function of inhibitory GABAergic interneurons (Truini et al. 2013; O’Connor et al. 2008). This latter notion is supported by the fact that spontaneous and evoked pain sensations in patients with spinal cord lesions including those due to MS are significantly reduced by intrathecal administration of baclofen, a GABA_B_ receptor agonist (Herman et al. 1992).

The pathogenesis of MS-associated TN is poorly understood. In an MRI study in 50 patients with TN, demyelination of primary afferents at the trigeminal nerve root entry zone was observed irrespective of whether TN was caused by MS, persistent neurovascular compression or a benign tumour (Cruccu et al. 2009). Primary afferent demyelination leads to the generation of ectopic impulses and paroxysmal pain (Burchiel 1980; Cruccu et al. 2009). Additionally, ephaptic spread secondary to juxtaposition of demyelinated axons and cells within MS plaques that release pro-inflammatory cytokines, likely also contributes to the pathogenesis of TN in MS (Love et al. 2001; da Silva et al. 2005). Based on MRI studies, the underlying mechanism for L’hermitte’s sign appears to be due to a negative impact of the demyelinating plaque on the dorsal column of the cervical spinal cord (Al-Araji and Oger 2005; Gutrecht et al. 1993).

Pro-inflammatory cytokines induce permeability changes in the blood–brain barrier to facilitate leukocyte infiltration and neuroinflammation (Holman et al. 2011; Minagar and Alexander 2003) that contribute to the pathobiology of MS-associated neuropathic pain (Sachs and Teixeira 2008; Boddeke 2001). In MS, dysregulated glutamate homeostasis involving downregulation of glutamate transporters, augmented glutamatergic neurotransmission via ionotropic glutamate receptors and upregulation of metabotropic glutamate receptors (mGluRs), contribute to demyelination, axonal damage (Werner et al. 2001; Sarchielli et al. 2003; Newcombe et al. 2008) and the pathogenesis of neuropathic pain (Osikowicz et al. 2013).

## Experimental autoimmune encephalomyelitis (EAE) rodent models of MS

Induction of EAE in rodents is a widely used approach for investigation of the pathophysiological mechanisms involved in the MS clinical disease course and for the evaluation of potential novel therapeutics for disease management (Gold et al. 2006).

Insights on the pathogenesis of MS gained through use of EAE-animal models, has been pivotal for the development of treatments that reduce MS disease relapses and attenuate disability (Constantinescu et al. 2011; Wilbanks 2012). Such treatments include glatiramer acetate, mitoxantrone, natalizumab, as well as the orally active drugs, fingolimod and teriflunomide, that were approved by the US Food and Drug Administration (FDA) in 1996, 2000, 2006, 2010 and 2012, respectively (Wilbanks 2012).

Comprehensive reviews on the history and use of EAE-animal models in MS research may be found elsewhere (Baxter 2007; Gold et al. 2006). In brief, EAE in animals has many features in common with MS in patients such as pattern of the clinical disease course, histopathological CNS lesions characterised by perivascular cuffs with mononuclear cell infiltration, gliosis, demyelination and axonal damage (Schreiner et al. 2009).

Experimental autoimmune encephalomyelitis can be induced reliably in a range of species including mice, rats, guinea pigs and rhesus monkeys by immunisation with various myelin proteins or their synthetic encephalitogenic components such as myelin oligodendrocyte glycoprotein (MOG), myelin basic protein (MBP) and proteolipid proteins (PLP) (Baxter 2007). The disease phenotype and histopathological features exhibited by each EAE-animal model depend upon the species, the specific myelin antigen and the immunisation protocol utilised (Constantinescu et al. 2011; Gold et al. 2006). For example, chronic-progressive EAE is induced in C57BL/6 and relapsing-remitting disease in SJL/J mice by immunisation with MOG_35–55_ and PLP_139–151_ peptides, respectively, emulsified with complete Freund’s adjuvant (CFA) (Constantinescu et al. 2011). However, C57BL/6 mice develop relapsing-remitting EAE disease using MOG_35–55_ as the antigen, but replacing CFA by saponins as the adjuvant (Peiris et al. 2007). Hence, the EAE disease course is also dependent upon the type of adjuvant used in the immunisation protocol. The distinct disease courses produced by various EAE-rodent models are invaluable for mechanistic investigation of specific MS disease phenotypes and for the assessment of novel treatments targeted to a particular phenotype (Simmons et al. 2013; Gold et al. 2006). The antigens used for disease induction together with a brief description of the associated clinical disease courses and respective histopathological features for commonly used EAE-rodent models are summarised in Table 2.

**Table 2:** EAE-rodent models: strain, encephalitogen and clinical disease course

Species	Strain	Encephalitogen	Clinical/pathological features	Reference
Mice	C57BL/6	MOG_35–55_	Chronic-progressive inflammatory demyelinating disease	Mendel et al. (1995)
		MOG_35–55_	Inflammatory demyelinating disease with rMOG_35–55_ whereas hMOG_35–55_ produced mild inflammation with no CNS demyelination	Albouz-Abo et al. (1997)
		MOG_35–55_	RR disease course characterised by CNS inflammation and demyelination	Peiris et al. (2007)
		PLP_178–191_	Milder disease relative to MOG-induced EAE in BL6 mice	Li et al. (2011)
	SJL/J	MOG_92–106_	RR with mild CNS demyelination	Tsunoda et al. (2000)
		PLP_139–151_	Severe RR disease course; extensive CNS demyelination and inflammation	McRae et al. (1992)
	Biozzi AB/H	MBP_12–26_	Mild EAE disease; subpial and perivascular infiltrates in CNS	Amor et al. (1996)
		PLP_56–70_	Chronic relapsing; demyelination and infiltrates in the CNS	Amor et al. (1993)
	NOD/Lt	MOG_35–55_	RR with cellular infiltration and multifocal CNS demyelination	Slavin et al. (1998)
	SJL.B	MOG_35–55_	RR disease course	Li et al. (2011)
Rats	Lewis	Gp and rMBP_68–88_	Gp MBP was more encephalitogenic with increased T-cell proliferation and circulating antibodies relative to rMBP	Kibler et al. (1977)
		Bovine PLP	Mild clinical disease; significant CNS demyelination	Yamamura et al. (1986)
		MOG_1–20_; MOG_35–55_	CNS inflammation without demyelination	Adelmann et al. (1995)
		MOG_1–125_	Mild disease with acute inflammatory demyelination	Adelmann et al. (1995)
	Dark Agouti	MOG_1–125_	Chronic and/or relapsing disease with demyelination	Weissert et al. (1998)
		SCH	RR characterised by demyelinating inflammatory lesions in spinal cord, T-cell infiltrates and perivascular evidence of immunoglobulins and complement	Lorentzen et al. (1995)
	Brown Norway	MOG_1–125_	Acute clinical disease course; significant CNS demyelination and inflammation	Stefferl et al. (1999)

*MOG* myelin oligodendrocyte glycoprotein, *MBP* myelin basic protein, *PLP* proteolipid protein, *SHC* spinal cord homogenate, *CNS* central nervous system, *EAE* experimental autoimmune encephalomyelitis, *Gp* guinea pig, *r* rat, *rMOG35-55* MOG of rat sequence, *hMOG35-55* MOG of human sequence, *RR* relapsing-remitting

Research on the biological processes involved in remyelination often uses toxins such as cuprizone, lysolecithin or ethidium bromide to induce demyelination in a range of species (Blakemore and Franklin 2008). Additionally, mice infected with Theiler’s murine encephalomyelitis virus (TMEV) are used to recapitulate CNS demyelination and other pathological features of MS (Tsunoda and Fujinami 1996). However, as the majority of research on the pathobiology of MS-associated neuropathic pain has used EAE-rodent models, the following sections of this review are focussed on insights gaining using EAE-rodent models to date (Handel et al. 2011).

## Pathobiology of MS-neuropathic pain: insights from EAE-rodent models

**Fig. 1:**
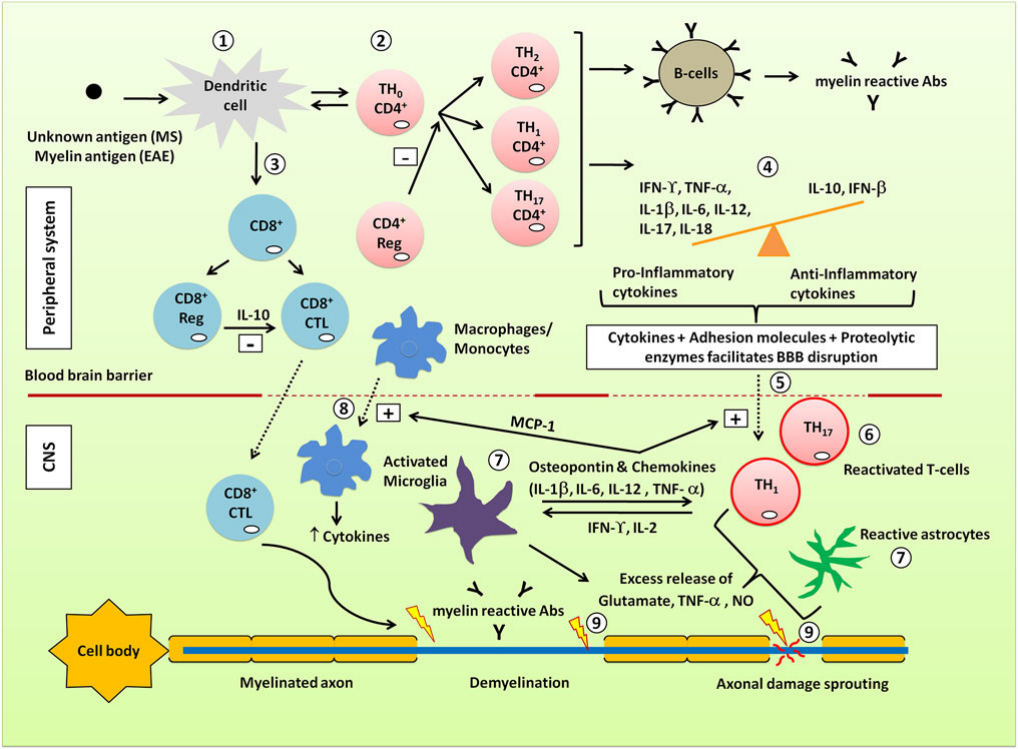
Pathophysiological cascades implicated in neurodegeneration associated with MS. (*1*) Unknown antigen (in MS) or myelin antigen (in EAE-animal models) is presented by dendritic cells to CD4^+^ T-cells. (*2*) In response, various subsets of CD4^+^ T-cells are activated and proliferate. (*3*) Primed CD4^+^ T-cells also cause dendritic cells to activate CD8^+^ T-cells resulting in clonal expansion of CD8^+^ regulatory (reg) T-cells and cytotoxic T lymphocytes (CD8^+^ CTL). CD8^+^ CTL can lead to direct damage of myelin sheaths and axons. Regulatory T-cells have a significant role in mitigating inflammatory processes. (*4*) Pro- and anti-inflammatory cytokines are released by activated T-cells along with activation of B-cells producing myelin reactive antibodies. (*5*) The blood–brain barrier (BBB) is compromised by interaction of cytokines secreted by activated T-cells with adhesion molecules (e.g. VCAM-1) on the surface of endotheliocytes together with matrix metalloproteases activity. (*6*) In the CNS, T-cells are reactivated to augment the inflammatory cascade. (*7*) Consequently, glial cells (microglia, astrocytes) in the CNS are also activated leading to excessive release of pro-inflammatory cytokines and excitotoxic substances e.g. glutamate, nitric oxide (NO). (*8*) Microglial expression of cytokines and the chemokine CCL2 [also called monocyte chemo-attractant protein-1 (MCP-1)] enhances infiltration of T-cells and macrophages, respectively. (*9*) These cascades lead to myelin sheath damage and axonal loss that underpin neuronal hyperexcitability and development of central neuropathic pain associated with MS

**Fig. 2:**
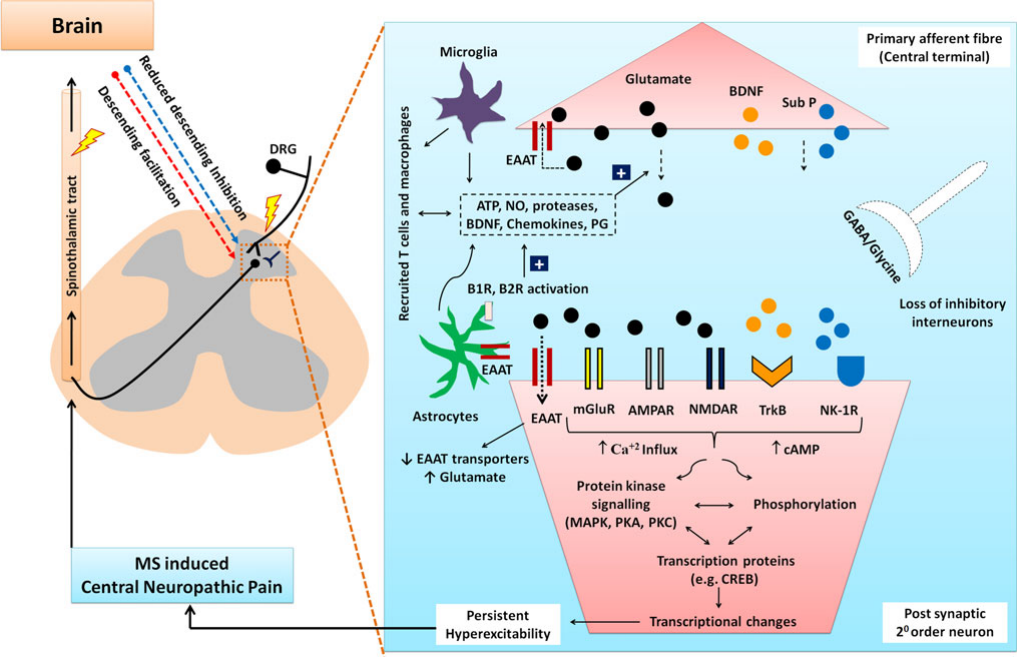
CNS pathobiological mechanisms implicated in MS-associated neuropathic pain

In EAE-rodent models of MS (Table 3), pro-nociceptive behaviours may be evoked reliably in the lower extremities, tail and hindpaws (Table 3), effectively recapitulating the predominantly lower extremity pain reported by patients with MS (Svendsen et al. 2005b). Hence, EAE-rodents are used for investigating the pathophysiology of MS-associated neuropathic pain. In addition, it may be possible to identify potentially ‘druggable’ targets for discovery of novel analgesics for improved relief of this chronic pain condition. In the following sections, progress to date is reviewed (Figs. 1 and 2).

**Table 3:** EAE-rodent models of MS-neuropathic pain studies

Species, sex/age or weight	Myelin antigen emulsified in CFA	Nociceptive assessments/drug treatments	Main findings	References
EAE-mouse models				
SJL/J ♂♀ 6–8 weeks	PLP_139–151_ (150 μg)	Thermal nociception in forepaws and tail (Hargreaves test)	Nociceptive changes prominent in tail; thermal hyponociception and hypernociception present during peak disease and recovery phase, respectively; more persistent hyperalgesia in female mice	Aicher et al. (2004)
C57BL/6 ♀ 8–10 weeks	MOG_35–55_ (100 μg)	Mechanical hypersensitivity in hindpaws (electronic von Frey device)	Mechanical hypernociception before onset of clinical motor deficits	Rodrigues et al. (2009)
C57BL/6 ♀ 10–12 weeks	MOG_35–55_ (50 μg)	Thermal nociception (Hargreaves test), cold allodynia (acetone test) and mechanical allodynia (von Frey filaments) assessed in hindpaws	Insignificant thermal hyperalgesia; robust cold and mechanical allodynia prior to and during onset of clinical disease; hyponociception during peak disease	Olechowski et al. (2009)
C57BL/6 ♀ 10–12 weeks	MOG_35–55_ (50 μg)	Noxious chemical stimulation due to intraplantar formalin injection in one hindpaw	Hyponociception in formalin-injected hindpaws of EAE-mice. Nociceptive responses normalised by treatment with MS-153 (glutamate transporter activator) or LY-341495 (mGluR2/3 antagonist)	Olechowski et al. (2010)
		*Drug treatments*:		
		MS-153 (10 mg/kg; I.P),		
		LY-341495 (0.25 mg/kg; I.P)		
SJL/J ♀ 8 weeks	PLP_139–151_ (100 μg)	Thermal (Hargreaves test); mechanical allodynia (von Frey filaments) assessed in hindpaws	Robust thermal hyperalgesia and mechanical allodynia in hindpaws of SJL/J mice during chronic disease; mild mechanical allodynia in hindpaws of C57BL/6 mice during onset and peak disease	Lu et al. (2012)
C57BL/6 ♀ 8 weeks	MOG_35–55_ (50 μg)			
C57BL/6 ♀ 7–8 weeks	MOG_35–55_ (400 μg) + Booster dose	Paw withdrawal latency to acute thermal nociception; (hot plate)	Thermal hyperalgesia in EAE-mice during disease onset and later phases of disease; OGF treatment reversed thermal hyperalgesia and attenuated disease progression in EAE-mice	Campbell et al. (2012)
		*Drug treatments*:		
		OGF (10 mg/kg; I.P)		
C57BL/6 ♀ 10–12 weeks	MOG_35–55_ (100 μg)	Mechanical allodynia (von Frey filaments) in fore- and hindpaws	Mechanical allodynia observed in both fore- and hindpaws before onset of motor deficits; Box5 and indomethacin alleviated mechanical allodynia	Yuan et al. (2012)
		*Drug treatments*:		
		Wnt5a antagonist Box5 (10 μg; I.T) Indomethacin (100 μg; I.T)		
C57BL/6 ♀ 8–12 weeks	MOG_35–55_ (300 μg) + Booster dose	Mechanical allodynia (von Frey filaments) in hindpaws	Mechanical allodynia prior to clinical disease onset; Rapamycin significantly reduced mechanical allodynia in hindpaws of EAE-mice and prevented development of clinical and histological signs of EAE disease when given prophylactically	Lisi et al. (2012)
		*Drug treatments*:		
		Rapamycin (1 mg/kg; I.P)		
C57BL/6 ♀ 10–12 weeks	MOG_35–55_ (50 μg)	Mechanical allodynia (von Frey filaments) in hindpaws. Association of nociceptive behaviour with cognitive deficits in EAE-mice also investigated	Robust mechanical allodynia prior to and during clinical disease onset. Development of cognitive impairment concurrently with mechanical allodynia in hindpaws Ceftriaxone attenuated mechanical allodynia and cognitive deficits	Olechowski et al. (2013)
		*Drug treatments*:		
		Ceftriaxone (200 mg/kg; I.P)		
C57BL/6 ♀ 6–10 weeks	MOG_35–55_ (200 μg)	Tactile allodynia (von Frey filament) and thermal hyperalgesia (hot plate)	Attenuation of mechanical allodynia by DALBK, a selective B_1_ receptor antagonist; abolished in B_1_R^−/−^mice. Mechanical allodynia exacerbated by DABK, a selective B_1_ receptor agonist. Thermal hyperalgesia reduced by HOE-140 (selective B_2_ receptor antagonist); abolished in B_2_R^−/−^mice	Dutra et al. (2013)
		*Drug treatments*:		
		des-Arg^9^-[Leu^8^]-BK (DALBK) (50 nmol/kg; I.P) des-Arg^9^-BK (DABK) (300 nmol/kg; I.P) HOE-140 (150 nmol/kg; I.P)		
C57BL/6 ♀ 12–16 weeks	MOG_35–55_ (100 μg)	Mechanical hyperalgesia (dynamic plantar aesthesiometer), cold allodynia (acetone test); heat hyperalgesia (hot plate) in hindpaws	Nociceptive testing before clinical disease onset; significant decrease in mechanical hyperalgesia and cold allodynia in CXCR3^−/−^ EAE-mice; also decreased EAE disease severity. Insignificant decrease in pain endpoints in *plt/plt* (CCL19/21) deficient mice	Schmitz et al. (2013)
EAE-rat models				
Lewis ♂♀ 8–10 weeks	Whole spinal cord (30 % suspension) or MBP (50 μg)	Vocalisation in response to noxious mechanical stimuli (applying pressure between thumbnails) in tail	No vocalisation in response to noxious mechanical stimuli applied to tail concurrent with ascending tail paralysis	Pender, (1986a)
Lewis ♀ (200 g)	SCH	Thermal hyperalgesia (hot plate)	Thermal hyperalgesia and motor deficits improved during chronic demyelinating phase of the disease by chronic administration of an [ACTH]_4–9_ analogue (neurotrophic peptide)	Duckers et al. (1996)
		*Drug treatments*:		
		[ACTH]_4–9_ analogue (75 μg/kg; S.C)		
Lewis ♂ 7–8 weeks	MBP (100 μg)	Nociceptive behaviours not recorded	Increased CREB phosphorylation in spinal cord of EAE-rats during peak disease implicated in pathogenesis of neuropathic pain	Kim et al. (2007)
Dark Agouti ♂ (250–300 g)	MOG_1–125_ (35 μg in IF)	Mechanical allodynia (von Frey filaments) in hindpaws	Hyponociception observed prior to onset of motor symptoms. During remission phase, robust mechanical allodynia in hindpaws. IL-10 gene therapy alleviated mechanical allodynia and significantly reduced paralysis	Sloane et al. (2009)
		*Drug treatments*:		
		pDNA-IL-10^F129S^ (plasmid DNA containing IL-10 gene); I.T		
Dark Agouti ♂ (250–300 g)	MOG_1–125_ (10 or 8.75 μg in IF)	Mechanical allodynia (von Frey filaments) in hindpaws	Significant mechanical allodynia before onset of motor deficits. Ceftriaxone reversed mechanical allodynia in EAE-rats and attenuated hind limb paralysis	Ramos et al. (2010)
		*Drug treatments*:		
		Ceftriaxone (150 μg; I.T)		
Lewis ♀ 5 weeks	MBP (500 μg) (Classical EAE) or MBP (500 μg) + cyclosporine (EAE + cyclosporine)	Mechanical allodynia (von Frey filaments), dynamic mechanical allodynia (paint-brush test), mechanical hyperalgesia (pinch test), cold allodynia/hyperalgesia (cold plate or 2 °C ice-cooled water), heat allodynia (42 °C water bath); hindpaws or tail	Dynamic mechanical allodynia not observed; other nociceptive behaviours were mostly robust and persistent over EAE disease course in rats administered cyclosporine Gabapentin-alleviated mechanical hyperalgesia; duloxetine- and tramadol-attenuated cold allodynia and cold hyperalgesia. EAE disease course remained unaffected	Thibault et al. (2011)
		*Drug treatments*:		
		(used in EAE + cyclosporine group) Acetaminophen (300 mg/kg; I.P) Duloxetine (30 mg/kg; I.P) Gabapentin (45 mg/kg; I.P) Tramadol (20 mg/kg; I.P)		
Lewis ♀	MBP (100 μg)	Nociceptive behaviour not recorded.	Elevated TNF-α expression in DRGs; anterograde transport to spinal cord implicated in development of neuropathic pain behaviours in EAE-rats	Melanson et al. 2009)
Lewis ♀	MBP (100 μg)	Mechanical allodynia (von Frey filaments) in hindpaws; thermal nociception (Hargreaves test) in tail, fore- and hindpaws	Mechanical allodynia in hindpaws not observed. Thermal hypoalgesia in tail and paws of EAE-rats during disease onset that was correlated with elevated TNF-α expression in DRGs and spinal cord	Begum et al. (2013)

*I.P.* intraperitoneal, *I.T.* intrathecal, *S.C.* subcutaneous, *MOG* myelin oligodendrocyte glycoprotein, *MBP* myelin basic protein, *PLP* proteolipid protein, *SCH* spinal cord homogenate, *[ACTH]4-9* adrenocorticotrophic hormone analogue, *EAE* experimental autoimmune encephalomyelitis, *CFA* complete Freund’s adjuvant, *IF* incomplete Freund’s adjuvant, *OGF* exogenous opioid growth factor (also called met-enkephalin), *CREB* cyclic AMP response element-binding protein, *DRG* dorsal root ganglia, *TNF-*α tumour necrosis factor-alpha, *IL* interleukin, *g* grams, *mg* milligram, *kg* kilogram, *nmol* nano molar

### Demyelination

In humans, MS-associated neuropathic pain develops secondary to demyelination and plaque formation in the brain and spinal cord (Nurmikko et al. 2010). In EAE-rats induced with spinal cord homogenate, extensive CNS demyelination induces sensory abnormalities as well as hindlimb weakness and paralysis (Pender 1986b). These clinical signs are underpinned by hyperexcitability of demyelinated axons, ectopic impulses, ephatic activity and conduction block (Pender 1986b). In the CNS of MOG-induced EAE-rodents (Table 3), there is widespread demyelination (Storch et al. 1998) together with anti-MOG antibodies and MOG-reactive T-cells (Iglesias et al. 2001) effectively mimicking neuropathological changes in the CNS of patients with MS (Storch et al. 1998; Iglesias et al. 2001). However, altered nociceptive behaviour in MBP-induced EAE-rats is underpinned, at least in part, by demyelination of primary afferent Aδ-fibres in the sacral and coccygeal dorsal root ganglia (DRGs), dorsal roots and dorsal root entry zones (Pender 1986a). Using electrophysiological methods, changes in the functional properties of peripheral nerve fibres in PLP-induced EAE-mice have also been characterised (Lu et al. 2012). Hence, as MS-associated neuropathic pain in humans is thought to develop secondary to CNS neuropathological changes, MOG-induced EAE-rodent models more closely mimic the human condition.

### Axonal damage

The high prevalence of axonal damage in the CNS of EAE-rodents appears to involve direct damage induced by cytotoxic T-cells and myelin autoantibodies (Hans 2010). In humans with MS and rodents with MOG-induced EAE, axonal damage is present not only in active lesions in the white matter, but also in the normally appearing white and grey matter (Kornek et al. 2000; Herrero-Herranz et al. 2008). As axonal damage contributes to ectopic firing of primary afferent sensory nerve fibres in rodent models of peripheral neuropathic pain (Costigan et al. 2009; Moalem and Tracey 2006), axonal damage may also underpin ephatic activity of CNS sensory neurons to induce central sensitisation in the spinal cord of EAE-rodent models as well as patients with MS-associated neuropathic pain.

### Recruitment of T-cells and other innate immune cells

A role for various T-cell subsets including CD4^+^, CD8^+^ and regulatory T-cells (T_reg_) in the pathophysiology of MS that is recapitulated in EAE-rodent models is well established (Chitnis 2007; Huseby et al. 2012; Anderton and Liblau 2008). However, the extent to which various T-cell populations contribute to the aetiology of MS-associated neuropathic pain is largely unknown. Prophylactic treatment of EAE-mice with the immunosuppressant, rapamycin, not only attenuated the development of neuropathic pain behaviours, but also reversed clinical signs of EAE disease (Lisi et al. 2012). The mechanism appeared to involve inhibition of effector T-cells, augmentation of T_reg_ cells (Esposito et al. 2010) and/or inhibition of glial cell activation (Lisi et al. 2012). Hence, future research directed at elucidating the contribution of various T-cell subsets to the pathobiology of MS-induced neuropathic pain is warranted. Other innate immune cells that contribute to CNS neuroinflammatory processes in MS and in EAE-rodents include mast cells, macrophages, neutrophils, dendritic cells and natural killer cells (Gandhi et al. 2010). These may also contribute to the pathogenesis of MS-associated neuropathic pain (Moalem and Tracey 2006).

### Pro-inflammatory cytokine and chemokine signalling

Pro-inflammatory cytokines, particularly TNF-α, IFN-ϒ, IL-1β, IL-6, IL-12 and IL-18, contribute to disruption of the integrity of the blood–brain barrier (Minagar and Alexander 2003) and to induction of CNS neuroinflammation in MS as well as in EAE-rodent models (Imitola et al. 2005). Their pro-inflammatory effects are offset by the actions of anti-inflammatory cytokines such as IL-10 (Imitola et al. 2005). Pro-inflammatory cytokines have a pathogenic role in the development of neuropathic pain (Zhang and An 2007). Conversely, augmented synthesis of IL-10 in the spinal cord of EAE-mice secondary to intrathecal administration of plasmid DNA encoding IL-10, suppressed glial cell activation and reversed mechanical hypersensitivity in the hindpaws, a hallmark symptom of neuropathic pain (Sloane et al. 2009). In EAE-rats, increased expression levels of TNF-α in the DRGs during peak disease with insignificant TNF-α mRNA expression in the spinal cord (Melanson et al. 2009) implicate anterograde transport of DRG-derived TNF-α to the spinal cord in the development of central sensitisation (Melanson et al. 2009). In support of this notion, thermal hypoalgesia in the tail and limbs of EAE-rats during disease onset was correlated significantly with inflammation induced by elevated expression of TNF-α in the DRGs with anterograde transport to the spinal cord (Begum et al. 2013). Although anti-TNF agents failed to attenuate MS disease progression in clinical studies, this may have been due at least in part to TNF receptor-1 gene variants in patients with MS (Gregory et al. 2012). Potential benefit for relief of MS-associated neuropathic pain by treatment with TNF-α antagonists such as the TNF receptor-Fc fusion protein (etanercept) and anti-TNF monoclonal antibodies (adalimumab and infliximab) remains to be investigated.

In preclinical studies, evaluation of the efficacy of specific monoclonal antibodies for a range of pro-inflammatory cytokines and chemokines implicated in the pathogenesis of MS-associated neuropathic pain is warranted. The chemokine, CCL2 (also called monocyte chemotactic protein-1, MCP-1), (Mahad and Ransohoff 2003) and the pleiotropic cytokine, osteopontin (Imitola et al. 2005) facilitate recruitment of T-cells and macrophages into the CNS of EAE-rodents. Hence, therapeutic strategies aimed at inhibition of CCL2 and/or osteopontin signalling have potential as treatments for MS-associated neuropathic pain.

During active demyelination in MS, there are elevated numbers of CXCR3- or CCR5-positive mononuclear cells in the cerebrospinal fluid (CSF) relative to the peripheral circulation (Sorensen et al. 1999). A role for chemokine signalling via the chemokine receptor, CXCR3, in MS-associated neuropathic pain is supported by observations of reduced neuropathic pain behaviour in EAE-mice null for CXCR3, that was accompanied by attenuated EAE disease progression (Schmitz et al. 2013). However, work by others found no benefit on EAE disease progression (Lalor and Segal 2013).

### Glial cell activation

Considerable research on peripheral nerve injury-induced neuropathic pain implicates glial cell activation in the spinal cord associated with the development of central sensitisation and neuropathic pain (Zhuo et al. 2011; Scholz and Woolf 2007; Hald 2009). Activated glial cells in the spinal cord release pro-inflammatory cytokines, glutamate and nitric oxide that amplify CNS neuronal hyperexcitability in neuropathic pain (Zhuo et al. 2011; Scholz and Woolf 2007; Hald 2009).

In EAE-mice, reactive gliosis and neuroinflammation in the spinal cord developed concurrently with robust neuropathic pain behaviours in a manner similar to that for neuropathic pain of peripheral origin (Olechowski et al. 2009; Lu et al. 2012). The underlying mechanisms include release of pro-inflammatory cytokines (e.g. IL-Iβ, IL-6, TNF-α) and neurotoxic molecules such as nitric oxide and prostaglandins from activated glia in the CNS (Rasmussen et al. 2007; Ponomarev et al. 2005). Apparently, contradictory reports that activated astrocytes enhance (Wang et al. 2005) and suppress (Matsumoto et al. 1992) neuroinflammation in EAE-rodents, require further investigation (Miljkovic et al. 2011).

### Excitatory and inhibitory amino acid neurotransmission

#### Excitatory neurotransmission

Augmented excitatory glutamatergic neurotransmission in the spinal cord mediated by impaired excitatory amino acid transporter (EAAT) function and transduced via ionotropic (NMDA, AMPA/kainate) and metabotropic glutamate receptors (mGluRs), is implicated in the pathogenesis of neuropathic pain (Osikowicz et al. 2013) in patients with MS (Werner et al. 2001; Sarchielli et al. 2003; Newcombe et al. 2008) and in EAE-rodent models (Olechowski et al. 2010; Ramos et al. 2010). In EAE-mice, neuropathic pain behaviours were abolished by treatment with the glutamate transporter activator, MS-153, or the mGluR2/mGluR3 antagonist, LY-341495 (Olechowski et al. 2010). Similarly, treatment of EAE-animals with ceftriaxone that upregulates spinal cord EAAT-2 expression levels, attenuated neuropathic pain behaviours (Olechowski et al. 2013; Ramos et al. 2010) and cognitive deficits (Olechowski et al. 2013).

#### Inhibitory neurotransmission

Once-daily parenteral administration of [met^5^]-enkephalin (also called opioid growth factor, OGF), to EAE-mice at 10 mg/kg for 40 days commencing after emergence of clinical disease symptoms, attenuated both motor symptom severity and thermal hyperalgesia in the hindpaws (Campbell et al. 2012). Alleviation of motor and sensory (pain) symptoms, despite the very short half-life of [met^5^]-enkephalin in the systemic circulation, was accompanied by significantly reduced CNS demyelination and astrocyte activation as well as inhibition of T-cell proliferation (Campbell et al. 2012).

### Neuropeptides

The neuropeptides, calcitonin gene-related peptide (CGRP) and substance P (Sub-P) are markers of small-diameter sensory neurons (Latremoliere and Woolf 2009). Hyperexcitability of these sensory neurons leads to the development of central sensitisation and neuropathic pain (Latremoliere and Woolf 2009). In EAE-mice, CGRP regulates IL-17 expression to promote Th17 cell-mediated CNS inflammation (Mikami et al. 2012). However, a role for CGRP in the aetiology of MS-neuropathic pain is discounted as spinal cord expression levels did not differ significantly between EAE and control mice (Lu et al. 2012; Olechowski et al. 2009). Although Sub-P and its NK-1 receptor are positively linked to CNS inflammation in EAE-mice (Reinke et al. 2006), a role for Sub-P signalling via the NK-1 receptor in the pathobiology of MS-neuropathic pain remains to be investigated.

### Bradykinin signalling

In patients with MS, bradykinin signalling via the B_1_ receptor has been proposed as an index for disease activity (Prat et al. 2005). In EAE-mice, bradykinin signalling via both B1 and B2 receptors is implicated in the pathogenesis of MS-neuropathic pain. Specifically in EAE-mice, mechanical hypersensitivity in the hindpaws was alleviated by treatment with a B_1_ receptor antagonist and abolished by genetic deletion of the B_1_ receptor, whereas treatment with a B_2_ receptor antagonist alleviated heat hyperalgesia in the hindpaws (Dutra et al. 2013). In EAE-mice, augmented bradykinin signalling was associated with increased expression levels of cyclo-oxygenase-2 (COX-2) and nitric oxide synthase-2 (NOS-2) (Dutra et al. 2013). The net result was upregulated endogenous production of prostaglandins and nitric oxide, respectively, as well as increased expression levels of the pro-inflammatory cytokines, IFN-ϒ and IL-17 (Dutra et al. 2013).

### Protein kinase signalling, phosphorylation and transcriptional changes

Increased phosphorylation of cyclic AMP (cAMP) response element-binding protein (CREB) by protein kinase-ϒ (PKC-ϒ) (Mao et al. 2007) that is co-localised with macrophages and astrocytes in the spinal cord of EAE-rats (Kim et al. 2007) is implicated in neuroinflammation in MS (Kim et al. 2007). In EAE-mice with severe hindlimb paralysis (chronic disability phase), there was a significant decrease in PKC-ϒ expression in the corticospinal tract (CST), whereas levels were normalised during the remission phase (limp or paralysed tail only) (Lieu et al. 2013). However, as spinal dorsal horn expression levels of PKC-ϒ did not differ significantly in EAE versus control mice, PKC-ϒ is unlikely to contribute significantly to the pathobiology of MS-neuropathic pain (Lieu et al. 2013).

Central sensitisation and long-lasting changes in synaptic plasticity in the dorsal horn of the spinal cord are mediated by phosphorylation of multiple receptors and ion channels by other kinases including p38 mitogen-activated protein kinase (MAPK) and p44/p42 MAPK (also known as ERK1/ERK2), protein kinase A (PKA), protein kinase G (PKG) and calmodulin protein kinase II (CaMK II) (Latremoliere and Woolf 2009). However, pharmacological manipulation of these pathways and their downstream effects on transcription factors such as AP-1 proteins, c-*fos*, and c-*Jun* in the aetiology of MS-associated neuropathic pain, remains for future investigation.

### Other potentially ‘druggable’ targets in MS-associated neuropathic pain

Neurotrophins including nerve growth factor (NGF) and brain-derived neurotrophic factor (BDNF), signalling via the tropomyosin-related kinase A receptor (TrkA) and tropomyosin-related kinase B receptor (TrkB), respectively, are implicated in the pathogenesis of peripheral neuropathic pain conditions (Siniscalco et al. 2011). Although neurotrophins also regulate neuronal growth and myelination in the CNS (Kalinowska-Lyszczarz and Losy 2012), it is unclear whether they have a protective or detrimental effect in MS-associated neuropathic pain. In a demyelinating EAE-rat model of MS, altered thermal nociception and motor deficits were improved by chronic treatment with the adrenocorticotrophic hormone analogue, [ACTH]_4–9_, suggesting that neurotrophic therapy may have benefit for relief of MS-associated neuropathic pain (Duckers et al. 1996).

A role for aberrant Wnt (wingless-type protein-1) signalling in the pathogenesis of MS-associated neuropathic pain is supported by observations of upregulated expression of the Wnt ligands, Wnt3a, Wnt5a and their downstream effector, β-catenin in the spinal dorsal horn in EAE-mice, with pain behaviours attenuated by treatment with the Wnt5a antagonist, Box5, or the β-catenin inhibitor, indomethacin (Yuan et al. 2012).

Apart from development of hindpaw hypersensitivity to applied mechanical and noxious heat stimuli in EAE-mice, most studies also report hyponociception (Table 3) particularly during disease onset (Pender 1986a; Aicher et al. 2004; Sloane et al. 2009) and at peak disease (Olechowski et al. 2009; Thibault et al. 2011). However, the validity of reports of hyponociception in EAE-mice has been questioned due to the potentially confounding effects of concurrent motor deficits (Table 3). Investigation of this issue in EAE-mice after intraplantar injection of formalin into one hindpaw showed that hyponociception appears to be underpinned by dysregulated glutamatergic signalling in the spinal cord (Olechowski et al. 2010). This was characterised by the downregulation of glutamate transporters resulting in increased synaptic levels of glutamate sufficient to activate presynaptic inhibitory group II mGluRs with the net result being attenuation of pain behaviours (Olechowski et al. 2010). In other work, thermal hypoalgesia in EAE-rats in the absence of motor deficits was correlated significantly with the extent of neuroinflammation and axonal swelling (Begum et al. 2013).

Collectively, research to date on MS-associated neuropathic pain using EAE-rodent models implicates complex neuroimmune mechanisms in the pathobiology of this condition. Multiple targets on CNS neurons and glial cells have been identified for potential use in novel analgesics discovery programs.

## MS-induced neuropathic pain: research gaps

### Clinical research

Optimal pharmacological management of CNP in patients with MS is a significant challenge for clinicians as there are very few randomised, double-blind, placebo-controlled clinical trials of analgesics or adjuvant drug treatments for alleviation of this condition. Most published clinical studies are limited by their open-label design, small patient numbers and relatively short duration (Table 1). Patients with MS suffer from multifaceted pain including neuropathic (e.g. ongoing extremities pain, trigeminal neuralgia or L’hermitte’s sign), nociceptive (e.g. pain arising from musculoskeletal problems) or mixed neuropathic, nociceptive pain (e.g. tonic painful spasms or spasticity) that involve distinct pathophysiological mechanisms (Truini et al. 2013). Although there are a few randomised double-blind placebo-controlled clinical studies of cannabis and low-dose naltrexone for the relief of MS-associated pain, the specific type of MS pain assessed in these patients was not specified (Wade et al. 2004; Sharafaddinzadeh et al. 2010).

Utilisation of standardised criteria that distinguish between the various pain components experienced by patients with MS and their respective responsiveness to analgesic/adjuvant drug treatments is needed to improve the conduct, reporting and value of such studies. In addition, changes to the design of clinical studies aimed at assessing the efficacy of disease-modifying treatments for not only reducing the rate of disease progression (relapses) in patients with MS, but also to include concurrent assessment of the effect of such treatments on the various components of MS-associated pain would be invaluable.

### Preclinical research

Apart from the paucity of RCTs assessing the efficacy of analgesic and adjuvant drug treatments for alleviation of MS-induced neuropathic pain, it is only relatively recently that research directed at elucidating the neurobiology of MS-induced neuropathic pain using EAE-rodent models has been undertaken.

A significant limitation of most studies using EAE-rodents in MS-associated neuropathic pain research is the use of CFA comprising heat-killed mycobacterium and paraffin oil, as the adjuvant to induce EAE disease (Gold et al. 2006; Stromnes 2006) (Table 3). This is problematic because administration of CFA alone increases expression of the pro-inflammatory mediator, COX-2 in the spinal cord and brainstem of control mice to match the levels in EAE-mice (de Lago et al. 2012). In addition, intraplantar injection of CFA in rats produced pain behaviours that correlated with elevated pro-inflammatory cytokine levels and glial cell activation in the CNS (Raghavendra et al. 2004). Hence, caution is required in the interpretation of pain behaviours in EAE-rodents when CFA is used as the adjuvant in the immunisation protocol. Replacement of CFA by adjuvants that do not themselves induce marked CNS neuroinflammation is a critical step in optimising EAE disease induction protocols in rodents for use in studies on the pathobiology of MS-neuropathic pain.

In EAE-rodents, the clinical disease course in most studies designed to gain insight into the mechanistic basis of MS-associated neuropathic pain includes periods of paralysis and motor impairment which preclude unambiguous assessment of behavioural pain endpoints at those times (Table 3). Thus, reliable assessment of pain behaviours is restricted to time points before motor impairment is evident. Hence, development of an optimised EAE-rodent model that exhibits robust neuropathic pain behaviours with a prolonged relapsing-remitting clinical disease time course, but without confounding motor impairment is needed. Such a model would facilitate a detailed investigation of the mechanisms underpinning the development and maintenance of neuropathic pain in a longitudinal fashion over the relapsing-remitting EAE disease course.

Myelin oligodendrocyte glycoprotein is a glycoprotein important to the structural integrity of the myelin sheath of neurons in the CNS (Baumann and Pham-Dinh 2001) and it is widely used as a powerful encephalitogen in EAE-rodents (Wekerle and Kurschus 2006; Mendel et al. 1995). MOG induces widespread demyelination and other pathological features in the CNS of EAE-rodents in a manner similar to that observed in patients with MS (Storch et al. 1998; Iglesias et al. 2001). Although EAE may be induced with either MBP or PLP, both of these proteins are found in the peripheral nervous system as well as the CNS. Hence, MOG is the preferred antigen as MS-associated neuropathic pain is regarded as being of central origin.

## Summary

For patients with MS-associated neuropathic pain, clinically available analgesics/adjuvant medications often provide inadequate pain relief. A contributing factor is that treatment evidence, derived from clinical trials of analgesic and adjuvant agents for the relief of MS-associated neuropathic pain to date, is limited by the open-label design, low patient numbers and the relatively short clinical trial duration (Table 1). Hence, well-designed, randomised, double-blind, placebo-controlled clinical studies are needed to assess the efficacy of drug treatments for the relief of the various dimensions of MS-associated neuropathic pain. In addition, knowledge on the pathobiology of MS-associated neuropathic pain is in its relative infancy compared with research on peripheral nerve injury-induced neuropathic pain. An urgent research priority is to establish an optimised EAE-rodent model of MS-associated neuropathic pain where the pro-inflammatory adjuvant, CFA, is replaced in the immunisation protocol by an adjuvant that itself does not induce CNS neuroinflammation or pain behaviours.

In summary, research to date implicates dysregulated glutamatergic signalling and glial cell activation in the CNS as key mechanisms in the pathogenesis of MS-associated neuropathic pain. Other potential mechanisms include enhanced bradykinin signalling via B1 and B2 receptors, upregulated Wnt signalling as well as augmented phosphorylation of CREB and other transcription factors in the CNS. Inhibition of pro-inflammatory cytokine signalling, augmentation of inhibitory cytokine signalling and/or blockade of chemokine receptor-mediated inflammatory cell recruitment to the CNS, have potential as future strategies for improving relief of MS-associated neuropathic pain.
